# Ion Conduction through the hERG Potassium Channel

**DOI:** 10.1371/journal.pone.0049017

**Published:** 2012-11-02

**Authors:** Luisa Ceccarini, Matteo Masetti, Andrea Cavalli, Maurizio Recanatini

**Affiliations:** 1 Department of Pharmacy and Biotechnology, University of Bologna, Bologna, Italy; 2 Department of Drug Discovery and Development, Italian Institute of Technology, via Morego 30, Genova, Italy; Dalhousie University, Canada

## Abstract

The inward rectifier voltage-gated potassium channel hERG is of primary importance for the regulation of the membrane potential of cardiomyocytes. Unlike most voltage-gated K^+^-channels, hERG shows a low elementary conductance at physiological voltage and potassium concentration. To investigate the molecular features underlying this unusual behavior, we simulated the ion conduction through the selectivity filter at a fully atomistic level by means of molecular dynamics-based methods, using a homology-derived model. According to our calculations, permeation of potassium ions can occur along two pathways, one involving site vacancies inside the filter (showing an energy barrier of about 6 kcal mol^−1^), and the other characterized by the presence of a knock-on intermediate (about 8 kcal mol^−1^). These barriers are indeed in accordance with a low conductance behavior, and can be explained in terms of a series of distinctive structural features displayed by the hERG ion permeation pathway.

## Introduction

**Figure 1:**
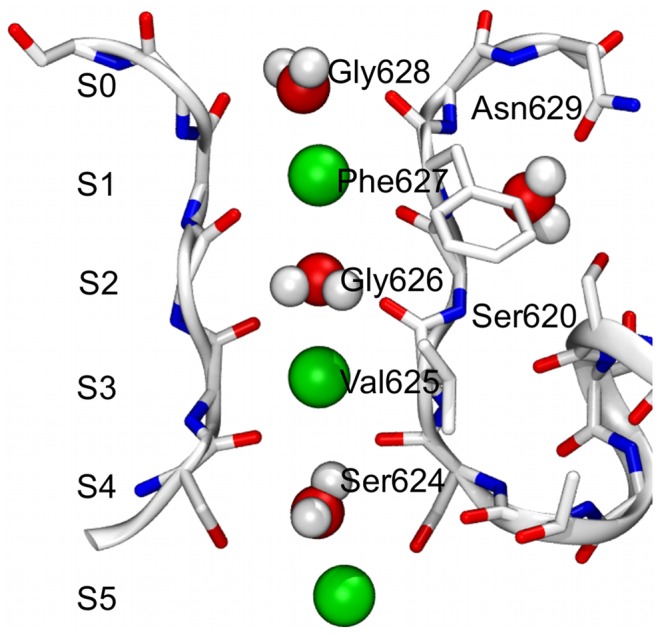
The Selectivity Filter of the hERG channel. The SF and the regions of the channel located in proximity of it are displayed as sticks and ribbons. For clarity, only two opposite subunits of the channel are shown. The depicted SF occupancy state (here referred to as S5[S3,S1]) involves three potassium ions and three water molecules (shown as van der Waals spheres). The additional water molecule placed behind the SF is also shown.

**Figure 2:**
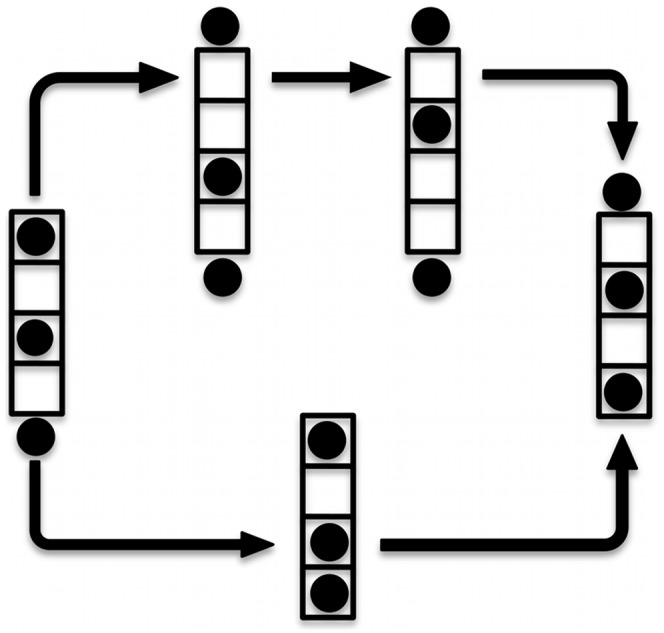
Schematic models of ion permeation. The ion permeation along a schematic selectivity filter displaying four coordination sites is shown. The cycle involves two opposite configurations of a doubly occupied filter (on the left and on the right), while a third ion is transferred from the intra- to the extra-cellular side. Only the rate limiting step of the whole permeation cycle is shown. The ion translocation can be achieved via both a knock-on (lower pathway) and a vacancy diffusion mechanism (upper pathway).

Potassium channels constitute a broad family of integral membrane proteins responsible for the passive permeation of K^+^ ions, whose functionality is of primary importance for any excitable cell [1]. Since the pioneering work of Hodgkin and Keynes [2], a remarkable effort has been made – both from an experimental and a theoretical standpoint – to unravel the mechanistic features responsible for both high selectivity and nearly diffusive conduction, typical of most K^+^-channels. Over the years, thanks to the always increasing availability of crystallographic structures and to the advancements in hardware and software technology, [3], the fundamental aspects of ion conduction through potassium channels have been eventually elucidated. It is now known that a key role in permeation is played by the Selectivity Filter (SF), the narrowest portion of the pore, where a sequential series of (at least four) coordination sites triggers potassium ions to adopt a single-file fashioned movement [4], [5]. Being K^+^-channels homotetramers, the SF is composed by four peptides, whose primary sequence (the so-called signature sequence: (T/S)XG(Y/F)G) is conserved among the whole protein family [6]. The backbone carbonyl oxygens of the SF, together with the hydroxyl oxygens of the threonine/serine, are arranged in such a way as to point toward the pore axis and form the vertices of the K^+^ binding sites, which are denoted as S1 to S4 starting from the extracellular side (Figure 1) [7], [8]. In good agreement with experimental evidence [2], [4], [8], computational methods predict an average occupancy of two K^+^ in the SF and an approximately unitary water/potassium flux ratio [9]. Taken together, these findings support the picture of a multi-ion conduction mechanism, where the translocation of adjacent ions is accompanied, on average, by an interposed water molecule. In spite of this, the modality by which the detailed translocation of ions through the SF is achieved is still a matter of debate. Most of the computational studies based on Molecular Dynamics (MD) simulations have confirmed the dominant view of a conduction based on the so-called knock-on mechanism [2], [5]. Such a mechanism is characterized by the formation of a metastable state in which a potassium ion that approaches the filter drives the conduction by forcing the SF to switch to the next occupancy state (Figure 2) [10]–[12]. Even though the energetics of this mechanism is consistent with the high throughput rate of conduction observed for typical K^+^-channels (up to 10^8^ ions per second [13]), alternative and competing mechanisms involving site vacancies [2], [14] have been reported to be accessible at room temperature [15].

To date, all the MD studies aimed at estimating quantitatively the energetic barriers governing the process of ion permeation through potassium channels have been carried out on high (or moderately high) conductance channels, such as the bacterial inward rectifiers KcsA [16] and KirBac1.1 [17], or the mammalian voltage-gated channel Kv1.2 [12]. To the best of our knowledge, no investigations on low conductance K^+^-channels have been carried out so far. Herein, the conduction mechanism of hERG, a low conductance voltage gated inward rectifier channel [18], [19], was investigated by means of a fully atomistic MD-based approach.

The hERG potassium channel (Kv11.1), encoded in humans by the ether-à-go-go-related gene KCNH2 [20], is expressed in several cells like cardiomyocytes, neurons, gastrointestinal smooth muscle myocytes, pancreatic cells, and tumor cells [21]. In cardiomyocytes, this channel is responsible for the rapidly activating delayed rectifier current (IKr), which is of primary importance for the membrane repolarization occurring after an action potential [22]. Due to the unique gating kinetics that originates from a voltage-dependent inactivation process that is faster than the activation, hERG exhibits outward currents of relatively small amplitudes at positive membrane potentials, and therefore it apparently behaves as a typical inward rectifier channel [23]–[25].

**Figure 3:**
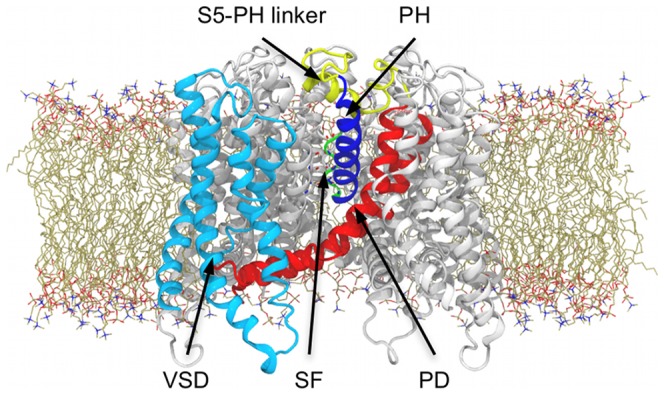
The simulated model system. The starting configuration for the complete (S1–S6) hERG model immersed in the DOPC lipid bilayer displayed as ribbons and sticks, respectively. For sake of clarity, water molecules and ions employed in the simulations are not shown.

No crystal structure for hERG is available yet; therefore we took advantage of one of the most reliable homology models available of the channel. This model was built using a state-of-the-art procedure driven by the available X-ray structure of the bacterial voltage-gated KvAP channel [26]), as well as NMR, and mutagenesis data [27]. According to this model, the overall architecture of hERG is consistent with that of most voltage-gated potassium channels: four α-subunits are co-assembled along the pore axis, to form the cavity and the SF. Three major domains can be distinguished: *i*) the voltage-sensing domain (helices S1–S4) responsible to monitor membrane potential changes, *ii*) the pore domain (helices S5–S6), that forms the channel lumen, and *iii*) the turret, a peculiar feature of this channel. As in all Kv channels, the SF is surrounded by the Pore Helices (PH), which directly interact with the filter residues and are supposedly implicated in its stabilization (see Figure 3).

Due to the aforementioned complex gating kinetics that involves several (and not fully characterized) conformational states [21], to study ion permeation in the most unbiased condition, an ideally active (*i.e.* open and conductive) conformation was employed throughout all simulations (see Methods for further details). To achieve a satisfactory statistics of ion conduction, microsecond long simulations should be performed [12]. However, a valuable and widespread alternative is to enhance the sampling of the conduction event along a reaction coordinate, that is typically defined as function of the position of the ions involved in the permeation.

In this study, the energetics of ions' translocation through the hERG SF was investigated by means of metadynamics [28], [29] together with a Path Collective Variable (PCV) approach [30]. Two different permeation pathways were identified: one at lower energy (about 6 kcal mol^−1^) consistent with a vacancy diffusion mechanism [2], [14], and the other at higher energy (about 8 kcal mol^−1^) that could be ascribed to a knock-on mechanism [2]. The reported result is in contrast with previous studies performed on high conductance channels, in which the knock-on intermediate has been identified as responsible for the low energetic barrier (2–3 kcal mol^−1^) of the transition [10], [12]. In the hERG channel, the distinctive molecular features of the conduction pathway might disfavor the knock-on intermediate and consequently raise the activation barrier. Notably, the relatively high energetic barrier found (6 kcal mol^−1^) is in fairly good agreement with the low conductance behavior of this channel.

## Methods

### System preparation

The lipid bilayer was built starting from a smaller pre-equilibrated patch [31], which was replicated until reaching an adequate size to include the model of the channel. Since no crystallographic data for the hERG channel is available yet, we used a homology model built by satisfying restraints derived both by sequence alignments and experimental data [26], [27]. The model comprises the voltage-sensing domain (S1–S4 helices), the pore domain (S5, PH and S6 helices) and whole sequence of the S5-PH linker (also known as turret; see Figure 3). Initial coordinates for the K^+^ ions and water molecules in the filter were taken from the crystallographic structure of KcsA (PDB ID: 1K4C [8]) upon superposition of the Cα atoms belonging to the SF residues of the two channels. Based on the coordinates of the KcsA channel, one additional water molecule per subunit was placed in the cavity between SF and PH (see Figure 1).

The system was fully solvated using the TIP3P water model [32]. A symmetrical ionic concentration of 0.1 M was used, and electroneutrality was reached adding 35 chloride ions and 40 potassium ions to the simulation box. The whole system comprised about 130,000 atoms.

An additional model was built by mutating the asparagine in position 629 of each subunit to aspartate (see below).

### Simulation details

All simulations were performed using NAMD-2.7 [33] patched with PLUMED-1.2.2 [34]. The Amber force field parm99SB [35] was used to model the protein. Lipids were treated with the General Amber Force Field [36], and missing parameters for the all-atom model of 1,2-dioleyl-sn-glycero-3-phosphorylcholin (DOPC) were taken by Rosso and Gould [31]. The recently re-derived ions parameters for the Amber force field [37], which were proven to better reproduce hydration free energies, were chosen.

All simulations were performed at 310 K (which is above the gel/Lα-liquid crystalline phase transition temperature for DOPC at the pressure of 1 atm [31]) by means of Langevin dynamics using a damping coefficient of 5 ps^−1^ and a uniform integration time step of 2 fs. Bonds involving hydrogen atoms were restrained to their equilibrium geometry with the SHAKE algorithm [38]. Short-range non-bonded interactions were treated using a cut-off radius of 10.0 Å together with a zero switching function active at distances larger than 8.0 Å. A neighbor list having a radius of 12.0 Å was used and updated every 10 integration time steps. Periodic boundary conditions were employed, and long-range electrostatics was estimated by means of the Particle-Mesh Ewald method [39] using a grid spacing of less than 1.0 Å in each dimension, and a fourth order spline interpolation scheme.

In order to prevent a complete unfolding of the uppermost portion of the filter, one additional water molecule per subunit was placed behind the SF. In particular, each water molecule was restrained to a distance of 3.0 Å with the δ oxygen of Asn629, and 2.9 Å with the γ oxygen of Ser620, using a spring force constant of 2.00 kcal mol^−1^ Å^−2^. Moreover, to avoid a possible incipient closure of the channel [40], a weak restraint of 1.00 kcal mol^−1^ Å^−4^ in the mean-square deviation (MSD) space was applied to helices S5, S6, and PH, using the initial geometry as reference frame.

The initial bilayer patch was equilibrated for 10 ns in the isothermal-isobaric ensemble using the Langevin Piston method as implemented in NAMD-2.7 [33], [41], [42] at the pressure of 1 atm using anisotropic pressure coupling. After insertion of the protein, the whole system was energy minimized using 5,000 steps of conjugate gradient. The system was then heated up to 310 K while positional restraints initially applied onto the protein atoms were smoothly released. At the end of the system equilibration, only the mild restraints above reported were preserved, and the system was further equilibrated for 1 ns. The root-MSD (RMSD) calculated over the protein backbone atoms along the 20 ns of equilibration shows that the channel exhibited an overall satisfying structural stability, as reported in Figure S1 in Supporting Information File S1. In particular the pore-domain and the voltage-sensing domain reached thermal stability after 2 ns.

All trajectories were analyzed with PLUMED-1.2.2 [34] and VMD [43].

### Collective variables

The detailed motion of three permeating ions in proximity and along the filter was explored by using PCVs together with a third collective variable (CV) suited to describe the folding state of the central portion of the SF.

PCVs consist of a pair of orthogonal variables designed to map the configurational space (**R**) of the atoms involved in their parameterization along a pre-defined path [30]:
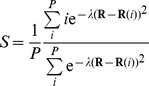


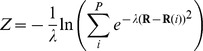
where *P* is the total number of *i* configurations employed in the definition of the path (in our case 19 configurations), and *λ* is a tunable parameter used to control the smoothness of the *S* function.

PCVs were parameterized so to encompass a complete cycle of permeation. Both the starting and the ending occupancy states of the cycle featured two SF-bound ions and were labeled as Sint,[S4,S2] and [S4,S2],Sext, respectively. In this notation, we include into the brackets the SF-bound ions and use the coordination site codes (*i.e.* SX) to specify the relative location of ions. Based on both previous studies on potassium channels and preliminary simulations, we designed an idealized path of ion motion where the 2-ions switches were preceded by the approach of a third ion. We described the permeation pathway with 16 frames. Accordingly, the main occupancy states Sint,[S4,S2], S5,[S4,S2], S5,[S3,S1], [S4,S3],S1, [S4,S2],S0, [S4,S2],Sext were mapped to *S* values of 0.00, 0.15, 0.25, 0.65, 0.85, and 1.00, respectively. The Cα atoms belonging to the SF residues were used as reference frame for the least square fit superposition. Conversely, the distance after superposition was measured using the position of the potassium ions only. The MSD metric was used to calculate the distance between the configurations describing the path, and the value of *λ* was set to 9.21 Å^−2^.

The folding state of the filter carbonyls was described with the *N_θ_* CV, which is a measure of the similarity of *n* dihedral angles to their reference values:
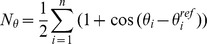



The values adopted by this CV range between 0 and *n*. An ideal value of *n* is returned when all the considered dihedral angles adopt their reference value.

Concerning the study of ion conduction, *N_θ_* was parameterized by taking into consideration the *ψ* angles of Val625 and Gly626 of each subunit, for a total of 8 dihedral angles. In this context, *N_θ_* is hereafter referred to as *N_S2,S3_* to keep it distinguished from *N_S1_* and *N_S4_* which are different CVs sharing the same functional form. Specifically, *N_S1_* and *N_S4_* were used to describe the folding state of the ion coordination sites S1 and S4, respectively. In particular, *N_S1_* was parameterized using the N-CA-C-O dihedral angles of Phe627, and *N_S4_* with the C-CA-CB-OG dihedral angles of Ser624 of each subunit. All the dihedral reference values were taken by the crystallographic structure of KcsA (PDB ID: 1K4C [8]).

### Metadynamics simulations

Two different simulation setups were employed for 3D and 1D metadynamics runs.

A 3D metadynamics simulation along the *S*, *Z* and *N_S2,S3_* CVs was performed to study ion conduction for both the WT channel and the Asn629Asp mutant by using the same path parameterization. In particular, the *N_S2,S3_* variable was required to accelerate the fluctuations of the filter's backbone atoms, which are thought to be essential to assist ion conduction [44] while avoiding at the same time the tendency to get trapped in a non-conductive conformation. For the WT simulation, the system was allowed to explore the whole space of *S*, whereas *Z* and *N_S2,S3_* were limited to values lower than 3.5 Å^2^ and larger than 5 units, respectively. The potassium ions were subjected to harmonic boundary potentials to keep them in the SF region, and to avoid the diffusion on the bulk. The Asn629Asp simulation was performed to investigate the effect of negative charges in proximity to the SF on the energetics of ion conduction. For comparison, only the CV space corresponding to the transition state regions of the WT was explicitly sampled on the mutant (*i.e.*, 0.2<*S*<0.9 and *N_S2,S3_*>7). The 3D metadynamics simulations were performed by adding a Gaussian biasing potential of 0.06 kcal mol^−1^ with the frequency of 0.5 ps^−1^. The Gaussian width was set to 0.2 units in both the *S* and *Z* dimensions, and 0.1 units for the *N_S2,S3_* variable. The multiple walkers approach of metadynamics [45] was used to optimally exploit parallel resources. In particular, ten walkers were run for an aggregate simulation time of about 200 ns.

To investigate the propensity of the S1 and S4 sites to preserve a K^+^ binding-competent conformation and consequently productive ion conduction, 1D metadynamics were performed along the collective variable *N_S1_* and *N_S4_* parameterized as described above. These simulations were performed by keeping the filter and the ions into the S5,[S3,S1] configuration. In the mono-dimensional case, a Gaussian deposition rate of 0.02 kcal mol^−1^ ps^−1^ with a CV width of 0.1 units was used.

### Brownian dynamics model

The BD model of ion permeation was performed within the formalism of a kinetic Monte Carlo algorithm where the instantaneous positions of ions were treated as a discrete state Markov chain, as described in [46]. In order to recover an unbiased 3D free energy as a function of the ion position along the Cartesian axis normal to the bilayer plane (in our case, the *z* axis) from a 2D space (the *S* and *Z* PCVs), a simple histogram reweighting scheme was applied.

The conductance of the channel was calculated by counting the number of productive ion translocation events along the simulated time at a given electrochemical potential. The simulation time ranged from about 3 to 5 µs. Details on the BD simulations parameters and the reweighting procedure are reported in Supporting Information File S1.

## Results and Discussion

The availability of an reliable homology model structure for hERG allowed us to perform MD-based simulations aimed at investigating both the mechanism and the energetics of ion conduction through this low conductance channel. The highly (virtually complete) conservation of the signature sequence (T/S)XG(Y/F)G) among the protein family [6] made us confident of the possibility to describe the leading molecular features involved in ion permeation at a fairly good level of description. As a matter of fact, this is not the first report on an investigation of the energetics of ion conduction through narrow pores relying upon a homology modeling-derived structure [47], [48]. However, at the best of our knowledge, this is the first computational study performed on a low conductance K^+^-channel.

### Mechanism of conduction

The energetics of a representative ion conduction cycle for the hERG channel was computed by means of metadynamics [28], [29] and path collective variables [30]. The investigated permeation cycle was designed to: *i*) involve a total number of three potassium ions; *ii*) allow a net transport of one K^+^ from the intracellular to the extracellular side; and *iii*) restore the initial SF occupancy state at the end of the permeation.

**Figure 4:**
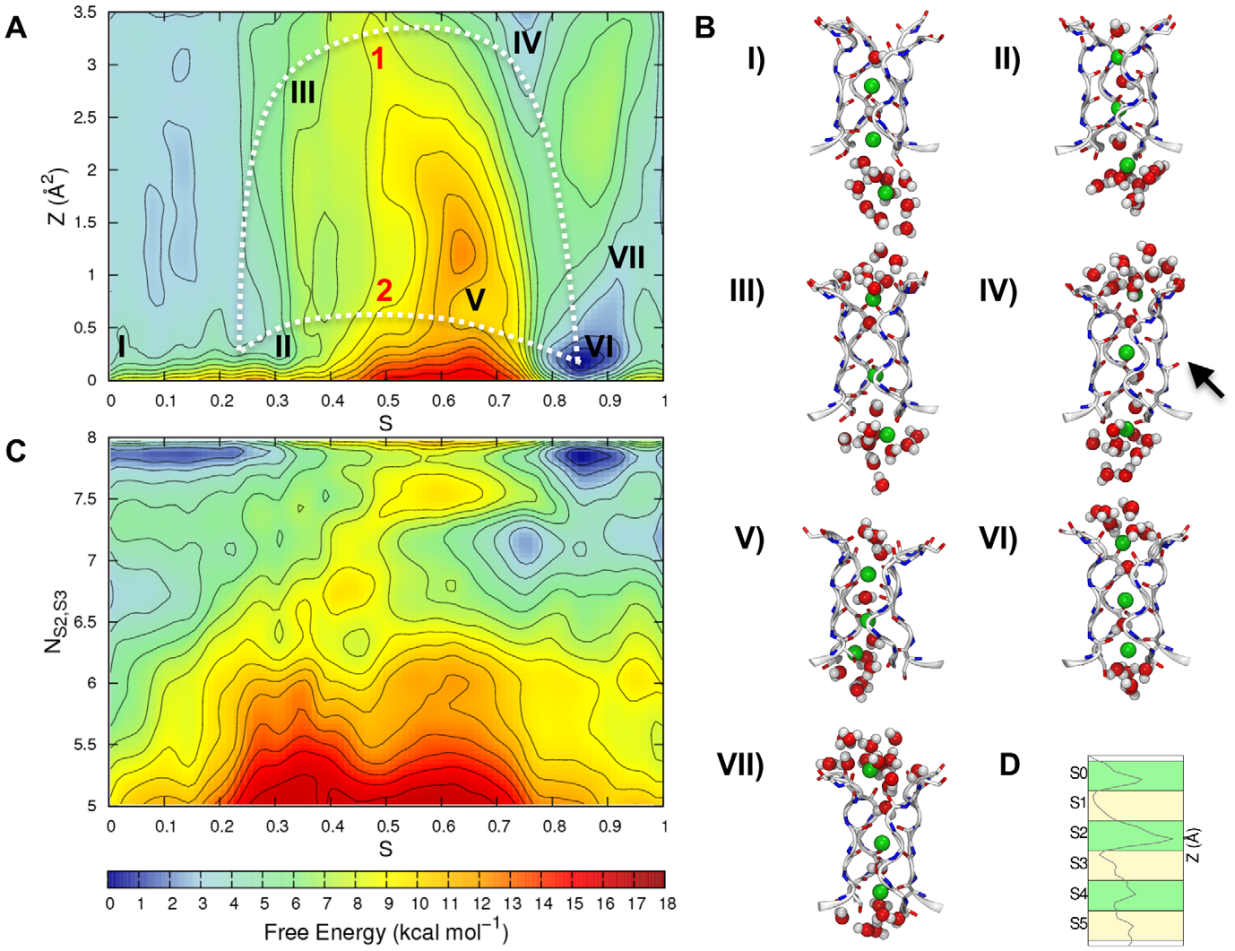
Energetics of ion conduction through the SF. Projections of the free energy as a function of *S* and *Z* (panel A), and *S* and *N_S2,S3_* (panel C) are shown as contour plots using an iso-line separation of 1 kcal mol^−1^. In panel A, the labels corresponding to the main basins found by the simulation (I to VII) as well as a schematic representation of the lowest energy pathways for the vacancy diffusion (pathway 1) and the knock on mechanism (pathway 2) are also shown. The representative configurations of the free energy basins are displayed in B. The arrow in the configuration corresponding to basin IV indicates a typical flipping of the Val625 carbonyl. D, representation of the K^+^ density of the filter averaged over the metadynamics trajectory.

2D projections of the free energy surface (FES) of ion conduction resulting from the 3D metadynamics simulation are shown in Figure 4 (a three-dimensional contour plot is also reported in Figure S2 in File S1). In particular, in Figure 4A, a 2D projection map calculated as a function of *S* and *Z* is reported. Seven central minima corresponding to as many discrete states could be distinguished, and are denoted as I to VII. In Figure 4B, configurations representative for each well are shown.

The conduction cycle started with *S* values close to 0.0, where basin I was located (Figure 4A). As expected, configurations belonging to this minimum displayed a Sint,[S4,S2] occupancy state, as shown in Figure 4B. In this SF configuration, the unbound K^+^ was quite free to move in the cavity of the channel. This was responsible for an asymptotic FES along the *Z* coordinate, revealing an important entropic contribution to the stability of the basin. A similar behavior was observed for minimum VII (*S* close to 1.0), where the initial SF occupancy state was restored and the permeation cycle closed, although in this case the unbound potassium ion was located in the external side of the membrane (occupancy state [S4,S2],Sext). The FES region with *S* between 0.0 and 0.4 corresponded to the approaching process of the innermost potassium toward the S5 binding site and the consequent translocation of the SF-bound ions, leading to the S5[S3,S1] state (basin II). The Sint,[S4,S2]→S5,[S3,S1] translocation turned out to be almost barrierless, in fair agreement with the previously reported studies performed on different potassium channels [10], [12], [15].

The central region of the FES (from basin II to basin VI) describes the switch between the two main occupancy states of the SF: S5,[S3,S1]→[S4,S2],S0, that is the entrance of a potassium in the SF from the inner cavity and the exit of the uppermost ion on the extracellular side. As shown in Figure 4A, at *S* between 0.4 and 0.8, the transition state region of the ion permeation process was identified. Accordingly, two different pathways could be distinguished at high (II→III→IV→VI) and low (II→V→VI) *Z* values in the PCVs space, labeled as pathway 1 and 2 in the figure (dotted white lines).

Pathway 1 was associated to the lowest energy barrier (about 6 kcal mol^−1^) and it was characterized by the unbinding of the uppermost potassium, S5,[S3,S1]→S5,[S3],S0 (transition from basin II to basin III), followed by the translocation of the incoming potassium within the filter, S5,[S3],S0→S5,[S2],S0 (transition from basin III to basin IV). Notably, the first step of this pathway led to the formation of a vacancy in site S2 (basin III), which shifted to site S4 in the following step (basin IV).This conduction mechanism is reminiscent of the so called vacancy diffusion, which has been identified as a secondary conduction pathway on high conductance channels such as KcsA and Kv1.2, in addition to the energetically more favorable knock-on mechanism [10], [12]. In our simulations, the knock-on mechanism was found at low *Z* values (pathway 2 in Figure 4A, transition II→V→VI), and it was associated to a higher energy barrier (about 8 kcal mol^−1^). Pathway 2 involved the formation of the knock-on intermediate [S4,S3,S1] (basin V), which was followed by the concerted translocation of the uppermost ions to the [S4,S2],S0 occupancy state.

In accordance with previous simulations on KcsA, the global minimum of the FES (VI) corresponded to the [S4,S2],S0 configuration. However, in hERG, the energy difference between the occupancy state [S4,S2],S0 and S5,[S3,S1] was found to be about 1 kcal mol^−1^ higher than KcsA [10].

As anticipated in the Methods section, the distinctive flexibility of the hERG SF prompted us to explicitly treat the conformations of the filter with an *ad hoc* CV. Indeed, during preliminary simulations, a pronounced tendency of the filter to partially unfold via the flipping of one or more SF carbonyls was observed. These unfolding events were found to be mostly irreversible over the limited timescales accessible by our MD simulations. Unfolded SF configurations were a sort of kinetic traps thus hampering a proper estimate of the FES for the ion conduction process. To accelerate the slow degrees of freedom associated to those transitions, the *N_S2,S3_* variable, describing in an average way the folding state of coordination sites S2 and S3, was used in addition to the already described PCVs. It is therefore particularly informative to observe the features of the FES projected along *S*, which describes the progression along the path, and *N_S2,S3_* (Figure 4C). As it can be seen, several metastable states located at *N_S2,S3_* lower than 7.5 (that correspond to misfolded SF conformations) could be identified. In particular, from Figure 4C, it can be observed that in correspondence of the occupancy states Sint,[S4,S2] (*S* corresponding approximately to 0.00), S5,[S4,S2] (*S*∼0.15), [S4,S2],Sext (*S*∼0.25), and, at a some lesser extent, S5,[S3,S1] (*S*∼1.00), the filter was found in an ideally conductive conformation (*N_S2,S3_* = 8) as well as in a partially unfolded state where one carbonyl was on average flipped (*N_S2,S3_*≤7). Most of the previously discussed minima in the *S*, *Z* space were found to lie in more than one folding state of the filter, even though the ideally conductive conformation turned out to be the most favorable rearrangement of the SF. The only exception was represented by the vacancy diffusion intermediates S5,[S3],S0 and S5,[S2],S0, which were mostly characterized by one flipped carbonyl (see Figure 4B, configuration III and IV). This is not surprising as it is well known that the conductive conformation of the filter is stabilized by the presence of the permeating ions. It is therefore reasonable that “unloaded”-K^+^ configurations of the filter such as S5,[S3],S0 and S5,[S2],S0 might display a flipped carbonyl.

By observing the 3D representation of the FES (see Figure S2 in File S1) it is also possible to notice that, while in pathway 2 (the knock-on mechanism) the transition occurred only at *N_S2,S3_* values close to 8, along the conduction pathway 1 (the vacancy diffusion) the transition might occur either with an ideally folded SF or with one carbonyl flipping. However, in the latter case the energy barrier was of about 2 kcal mol^−1^ higher than in the former, confirming the importance of folded coordination sites during ion translocation.

As previously mentioned, the SF sequence is highly conserved throughout the whole family of K^+^ channels, as it encodes for the physico-chemical features required to achieve a high potassium selectivity without significantly affecting the rate of conduction [10]. In spite of that, sequence deviations either on the SF or especially in proximity of it do occur, and together with the gating kinetics they are possibly involved in determining the distinctive conductance behavior of channels. In the following paragraphs, the peculiar features of the hERG SF and surrounding regions will be discussed with a particular emphasis on their difference with respect to the extensively studied KcsA channel, which is taken as a reference for a prototypical high conductance channel. At a molecular level, the hERG pore differentiates from that of KcsA in three main aspects: *i*) backbone flexibility of the SF, *ii*) electrical balance due to the net charge of the residues belonging to the SF or located in proximity of it, and *iii*) nature of the amino acids in the cavity at the entrance of the SF which are involved in the K^+^ desolvation.

### Filter flexibility

Among the several possible explanations, the unusually rapid inactivation kinetics of the hERG channel has also been attributed to the intrinsic instability of its selectivity filter [49]. An evidence supporting this hypothesis is the substantial SF flexibility previously observed [40] [50], when studying homology derived structures of hERG by means of MD simulations. The reason for this behavior might be found in the replacement of a tyrosine with a less conserved phenylalanine (at position 627 in hERG), which reduces the extension of the H-bond network in the surroundings of the filter [51]. Another potential source of weakness of the hERG SF compared to that of KcsA is the significant difference in amino acid composition of the residues located in the pocket at the interface between SF and PH (see Figure 1). While in KcsA a charge-assisted H-bond network involving the side chains of Glu71), Asp80, and the backbone of SF residues is established [52], in hERG all these interactions are missing. Instead, in the corresponding positions of those amino acids, Ser620 and Asn629 are found, which cannot be involved in favorable interactions with the filter backbone as in KcsA. As a further consequence of the different amino acid composition, in the hERG model a larger cavity is formed in the region behind the SF when compared to KcsA. The presence of a water molecule involved in the aforementioned H-bond network in the KcsA crystal structure, suggests that on average a higher number of solvent molecules should be found in the hERG channel. As previously reported [40], [50], [53], water molecules located in this pocket might play a relevant role in the dynamical stabilization of the SF. However, since determining their correct number is far from trivial, in line with previous works [40], only one water molecule was used to setup the calculations (see Methods). Notably, during simulations, a variable number of water molecules per subunit, on average ranging from 2 to 3, was reached. These peculiar features of the hERG channel might further contribute to the flexibility of the pore region, which in our study was especially experienced by the outermost moiety of the filter, namely the one involving residues ranging from Gly626 to Gln629. It is important to note that a similar behavior was obtained in previous works [40], [48] even though hERG model, force field, and lipid model used were different.

**Figure 5:**
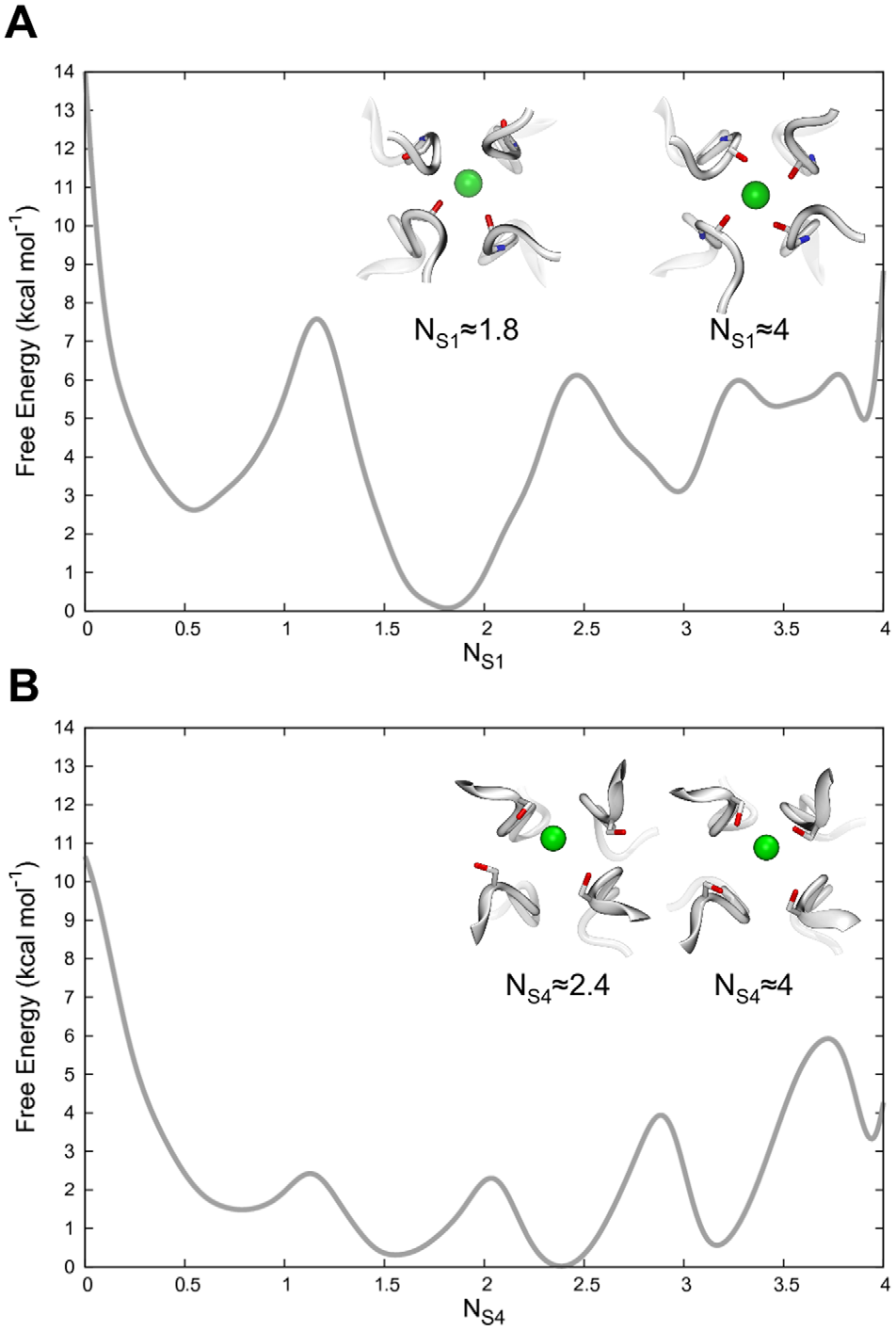
Stability of the coordination sites S1 and S4. Free energy profiles describing the stability of the coordination sites S1 and S4 as a function of *N_S1_* (panel A) and *N_S4_* (panel B), respectively. In both cases, the S5,[S3,S1] occupancy state of the filter was used. In the insets, representative configurations of the filter are shown.

According to the minimum energy path here observed (Figure 4A and B, pathway 1), the translocation of the uppermost SF-bound ion from the coordination site S1 to S0 preceded the binding of the incoming potassium at the S4 site (configurational transition II→III and IV→VI, respectively in Figure 4A and C), likely as a consequence of the flexibility of the outermost part of the filter. From the calculated K^+^ density averaged over the 3D metadynamics trajectory (Figure 4D), the low propensity of site S1 to keep bound a K^+^ ion appears evident. To confirm such a behavior in terms of a reduced intrinsic stability of the S1 site, a 1D metadynamics simulation was performed along the *N_S1_* variable. We note that the result of the calculation is a conditional free energy, since the occupancy state of the filter was purposely maintained in the S5,[S3,S1] configuration. As Figure 5A shows, the preferred conformation adopted by the backbone of the four Phe627 residues, displayed an average value of 2 folded carbonyls. Notably, the quite broad global minimum was 5 kcal mol^−1^ more stable than that corresponding to a completely folded S1 state (*N_S1_* approaching to 4).

As already discussed in the context of the conduction mechanism, a marked flexibility was also experienced by the central portion of the filter, in particular by the backbone of Val625 and Gly626. Specifically, the *ψ* angle of Val625 of one subunit was flipped along most of the vacancy diffusion pathway. Since these residues are not distinctive of hERG but they are rather shared with other channels such as KcsA, we are inclined to attribute the increased flexibility to a conformational transmission exerted either by the intrinsic S1 instability, or by a resulting effect due to the peculiar amino acid composition in proximity of the SF. It is interesting to note that the Val-Gly amide flipping in the corresponding KcsA residues has been hypothesized as a mechanism of intrinsic closure of the filter, the so called C-type or “slow” inactivation. Indeed, Roux and coworkers calculated an increase in the energy barrier of permeation of about 3 kcal mol^−1^ associated to this conformational change of the filter, leading to a virtually nonconductive channel [54]. We note on passing that this is a completely distinct phenomenon to the hERG inactivation, which is in fact known to be a “fast” process [25], [48]. Based on our simulations, we propose that during permeation the hERG SF might visit some partially unfolded states, which are distinctive of its permeation mechanism. Those misfolded SF configurations are predicted to be reversible, and should not to viewed as nonconductive states, since the associated energy barrier is in line with the low efficiency permeation pathway displayed by this channel.

### Electrical Balance

The large variability in conductance observed within the family of K^+^-channels has been associated to the electrical balance between ions in the filter and residues located in the surroundings of the SF, that is in the PH [55]–[57]. Accordingly, high conductance K^+^-channels show negatively charged residues 6–8 Å apart from the ion permeation pathway. In KcsA, this charge is provided by four carboxyl-carboxylate pairs formed by the sidechains of Glu71 and Asp80 [5], [8], [56], [57]. The resulting electrostatic field turns out to favor the presence of cations in the filter, thus balancing the ion-ion repulsion and allowing up to three K^+^ ions to be located in the SF, consistent with a knock-on mechanism of conduction. Conversely, like most of low conductance channels, hERG exhibits no negatively charged residues in the region surrounding the SF. In particular, in the positions corresponding to Glu71 and Asp80 in KcsA, hERG carries Ser620 and Asn629. The repulsion between ions in the filter is therefore not properly balanced by long-range electrostatic effects, and this might give rise to a conduction mechanism involving vacancies and consequently higher energetic barriers.

**Figure 6:**
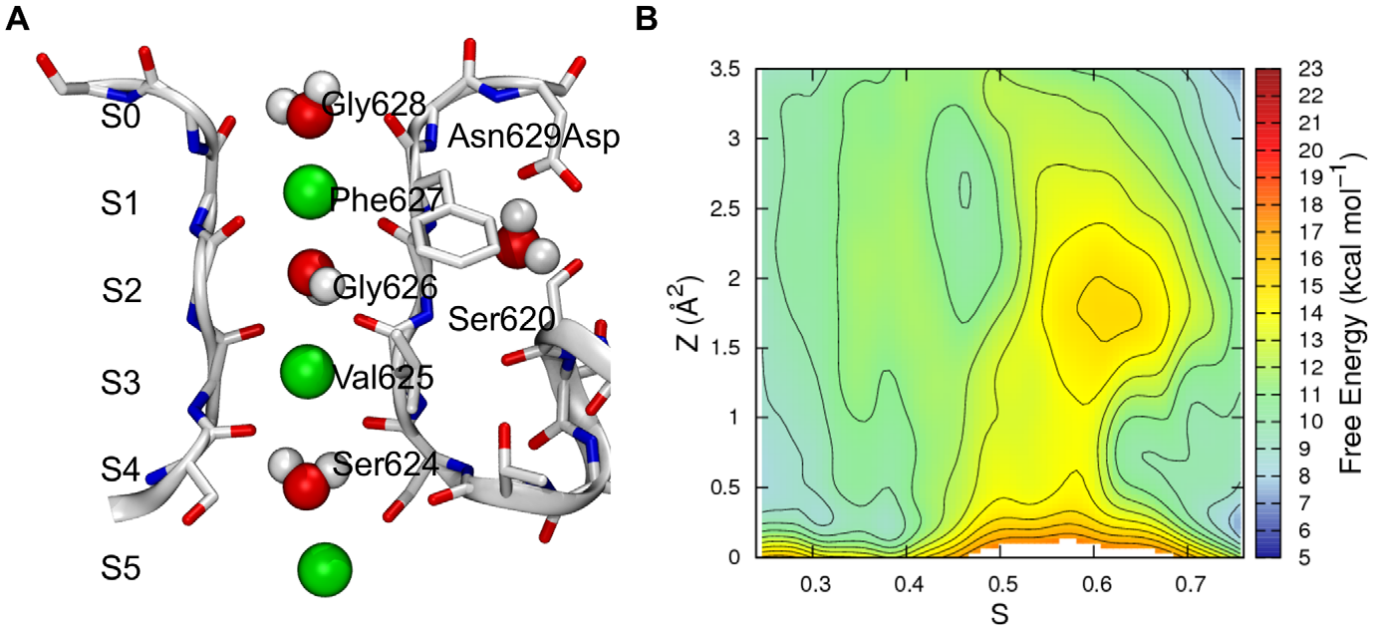
Effect of negatives charges behind the filter. A, the SF of the Asn629Asp mutant. B, Free energy of ion conduction as a function of *S* and *Z* calculated for the mutant. The free energy surface is shown as contour plot using an iso-line separation of 1 kcal mol^−1^.

To test such hypothesis, the transition state region of the ion conduction pathway (*S* approximately comprised between 0.4 and 0.8 units, and *N_S2,S3_* larger than 7), was also sampled for the Asn629Asp mutant (Figure 6A). In Figure 6B, we report the 2D-FES calculated for the mutant. The free energy surface was arbitrarily aligned onto the S5,[S3,S1] state. As it can be inferred by the plot, the presence of four negative charges in proximity of the SF reduced the energy barrier of both conduction mechanisms (about 1 kcal mol^−1^ for the vacancy diffusion mechanism, and 2 kcal mol^−1^ for the knock-on). Interestingly, in the mutant, the vacancy diffusion mechanism reached a barrier comparable with that calculated for the same mechanism in KcsA [10], whereas the knock-on mechanism still resulted to be less favorable. This result suggests that the electrical balance alone is not sufficient to provide both an inversion in the energetics of the two conduction mechanisms and, more importantly, a significant increase of conductance in this channel.

According to experimental evidence, the Asn629Asp mutation influences the hERG functionality at different levels, by altering both the gating and the selectivity of the channel [58]. These functional aspects of the mutant are not covered here, and further studies should be undertaken to properly address these features.

### Desolvation

The binding process that underlies the transfer of potassium from the aqueous solution to the protein environment (in this case the innermost SF site, *i.e.* S4) is a critical step for the whole permeation cycle, as it must satisfy the strict requirement to provide ionic selectivity along with a high rate of conduction [5], [8]. KcsA crystal structures [7], [8], and later a number of MD simulations [3], [9], [10], have contributed to rationalize the molecular mechanism responsible for bringing efficiently a K^+^ ion from its hydrated state in solution to its dehydrated state in the SF. The oxygen atoms forming the vertices of each SF binding site are well arranged in such a way as to mimic the first hydration shell of a potassium ion in solution. For sites S1 to S3, those oxygen atoms are provided by the carbonyl groups of the SF backbone, whereas S4 is the only site where half of the square antiprism vertices are formed by hydroxyl oxygen atoms belonging to the side-chain of a threonine (as in KcsA, Thr75) or a serine (as in hERG, Ser624). In order to optimally satisfy such geometry, the C-CA-CB-OG dihedral angle of the threonine/serine should adopt a value of approximately −60 degrees or, by using our CV notation, *N_S4_* should ideally reach a value of 4.

We speculate that inside the sterically hindered cavity of channels the propensity to adopt different side chain conformations is greater for serine than for the bulkier threonine. In other words, threonine might be better suited to assist the conduction by favoring the K^+^ desolvation and the concurrent binding to S4. For these reasons, the energetic cost of K^+^ desolvation might be higher in hERG compared to KcsA, and thus the simultaneous presence of three potassium ions in the SF (as required by the knock-on mechanism) unlikely. The lower tendency of site S4 to accommodate a potassium ion is clearly visible in the calculated K^+^ density averaged over the trajectory (see Figure 4D). Similar features were experimentally observed in the crystallographic structure of the Thr75Cys KcsA mutant, where the density profile of K^+^ ion was altered, showing a broader and shifted peak in site S4, consistent with lowered occupancy. A dramatic reduction of conductance for the mutated channel compared to the wild type was also reported [59]. Despite both chemical and volumetric differences between serine and cysteine, we suspect that deviations from the conserved threonine at this position of the SF might play a role in the fine tuning of the conductance of channels. To substantiate such hypothesis, a 1D metadynamics in the *N_S4_* space was performed. As already discussed for the stability of the coordination site S1, we stress that the result of the calculation has to be considered as a conditional free energy. The free energy profile as a function of *N_S4_* (Figure 5B) showed a global minimum in correspondence of a value of about 2.5 units. The completely folded S4 site was 3 kcal mol^−1^ less stable than the previous one, and the highest energy barrier separating these minima was about 6 kcal mol^−1^.

### A semi-quantitative estimation of conductance

Given the important role played by hERG currents in the cardiac repolarization, it is of considerable interest to attempt to relate the energy barriers obtained by atomistic simulations to the experimental conductance of the channel. As a matter of fact, accordance among the calculated and the actual conductance would be a direct confirmation of the observed conduction mechanism and, on top of that, of the reliability of the employed channel model. Unfortunately, in the case of the hERG channel, this task is complicated by the peculiar inactivation process experienced by the protein, which has a remarkable effect on the experimentally detectable currents. As already mentioned, the physiologically relevant hERG currents are outward K^+^ currents whose amplitude is considerably downsized by a voltage dependent inactivation process. Specifically, at depolarizing potentials, where these currents take place, a so far not completely understood conformational modification, promptly brings the channel in a nonconductive state. More precisely, inactivation becomes a sizeable effect at positive potentials larger than about +40 mV, when measured in 100 mM symmetric K^+^ concentrations [18], [19]. Conversely, no inactivation process is measured at negative potentials. For these reasons, hERG apparently acts as an inward rectifier channel, meaning that at the same absolute electrochemical potential, inward currents are larger in magnitude than outward currents [60].

Apart from the inactivation mechanism that is not explicitly covered here, the ion conduction pathway of the hERG channel appears to be significantly less efficient than that of more studied channels, such as KcsA. This has nothing to do with inactivation, and rather reflects a different microscopic behavior of the SF of the two proteins when the ion permeation is measured in consistent experimental conditions for an open and conductive conformation of the channels. An effective way to detect this occurrence is to compare the slope conductance (or zero-voltage conductance) of the two channels in the linear regime of the current-voltage (I-V) relationship. In symmetrical 100 mM K^+^ solutions, the slope conductance of KcsA is 97 pS [61], whereas values ranging from 9.7 [18] to 12.6 pS [19] in the inward direction were recorded for hERG. These mean that the KcsA filter has an ion conduction ability of about one order of magnitude higher than the hERG SF. In the outward direction, above the threshold of +40 mV, the I–V relationship for hERG is no longer ohmic because of the inactivation process, thus the even smaller conductance values recorded (chord conductance of 3.5 pS at +100 mV [18]) should not be used as comparison.

To test the reliability of the atomistic simulations, we attempted to estimate the conductance of the hERG channel model by using the calculated free energy of ion motion through the SF to feed a BD simulation (see Supporting Information File S1 for details). As a result, we obtained an average chord conductance of 5.1 pS at −40 mV, and 2.5 pS at +40 mV. These results indicate that the order of magnitude of the conductance is correctly predicted by our model. However, a non-negligible mismatch between actual and calculated values, most likely due to an overestimation of the energetic barriers by the atomistic simulations, is also evident. Given the many layers of complexity and the approximations employed to perform simulations (*i.e.*, homology model, force field, histogram reweighting, and the parameters employed for the BD simulation), we consider the observed vs. predicted agreement acceptable, even though not yet conclusive.

### Conclusions

The rationalization of the mechanisms involved in the permeation of ions through narrow pores, such as the SF in potassium channels, has been a matter of interest since the pioneering works in electrophysiology, which date back to the middle of the past century. To explain the high ion transport efficiency shown by K^+^-channels two main mechanisms of conduction, both apparently consistent with experimental data, have been proposed: the already discussed knock-on [2] and the vacancy diffusion [2], [14] models. The latter differs from the more popular knock-on in that ions are supposed to move through the SF sites via the formation of transient vacancies (being either represented by water filled sites or voids), which travel in the opposite direction of ion motion. The knock-on model has then emerged as a dominant conduction mechanism as it was observed by means of MD simulations by Roux and co-workers first [10], and later by other groups [11], [12], when studying the permeation of high conductance channels. However, it is reasonable that channels showing a different conductance in the linear current-voltage regime might exploit different conduction mechanisms as a result of specific energetic pathways encountered through the SF.

Here, the ion conduction mechanism of hERG, a low conductance channel was studied. The outcome of our investigation might be summarized as two major findings: i) two different mechanisms compete for hERG channel conduction, *i.e.*, knock-on and vacancy diffusion, being the second one the energetically favored; ii) in line with the previous result, a relatively high energetic barrier of 6 kcal mol^−1^ (against the 2–3 kcal mol^−1^ estimated for high conductance channels [10]–[12], [15]) was obtained. These findings are consistent with the low conductance behavior displayed by the channel, and might be attributed to a series of peculiar features that taken together render hERG unique in the family of voltage-gated potassium channels. By analyzing our data, those features were identified in: *i*) a peculiar flexibility of the filter, *ii*) the lack of negatively charged residues in proximity of the conductive pore and, *iii*) a different conformational behavior showed by Ser624 compared to the more conserved threonine residue at the same position of the bottom of the selectivity filter. In the light of our results, we can conclude that in low conductance channels such as hERG, the knock-on mechanism is most likely suppressed in favor of some less efficient mechanism, opening new perspectives to understand the complexity of these biophysical machineries.

## Supporting Information

File S1 File S1 contains Figure S1 describing the channel stability, Figure S2 showing a 3D representation of the free energy, and a detailed description of the reweighting procedure and the Brownian dynamics simulation.
(DOC)
