# Supplementary material for: Ion Conduction through the hERG Potassium Channel
Source: PLoS One. 2012 Nov 2;7(11):e49017. doi: 10.1371/journal.pone.0049017 (PMC3487835; doi:10.1371/journal.pone.0049017)
Supplement: File S1 — contains Figure S1 describing the channel stability, Figure S2 showing a 3D representation of the free energy, and a detailed description of the reweighting procedure and the Brownian dynamics simulation. (DOC) [file pone.0049017.s001.doc]

**SUPPORTING INFORMATION**

**Ion conduction through the hERG potassium channel**

Luisa Ceccarini^1^, Matteo Masetti^1^*, Andrea Cavalli^1,2^, and Maurizio Recanatini^1^

^1^Department of Pharmacy and Biotechnology, University of Bologna, via Belmeloro 6, I-40126 Bologna, Italy

^2^Department of Drug Discovery and Development, Italian Institute of Technology, via Morego 30, I-16163 Genova, Italy

**Contents**

**Supporting figure S1 S2**

**Supporting figure S2 S3**

**Parameterization of the Brownian Dynamics model S4**

**References S6**


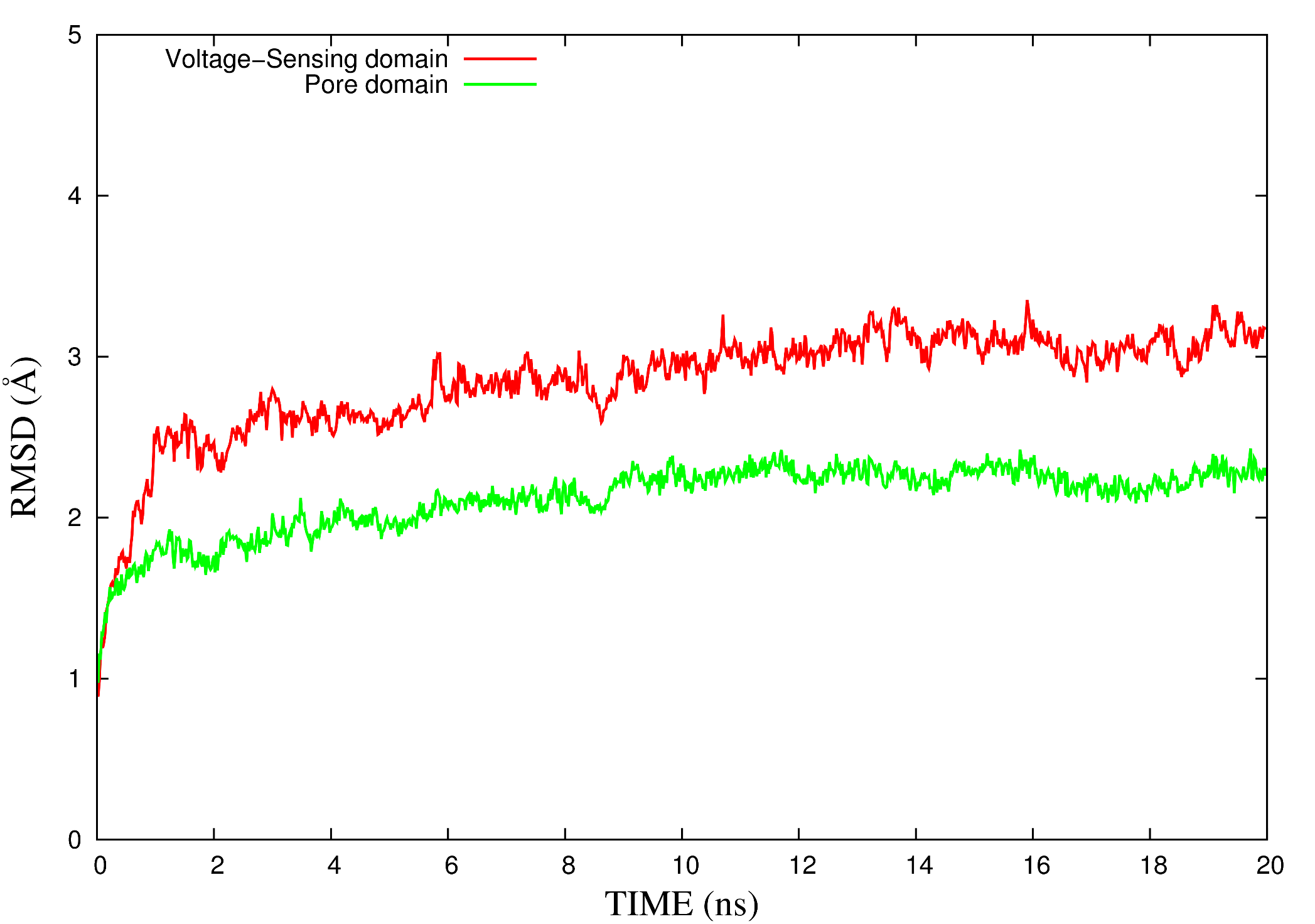


**Figure S1.** **Protein stability.** Backbone RMSD values plotted against 20 ns of equilibration.


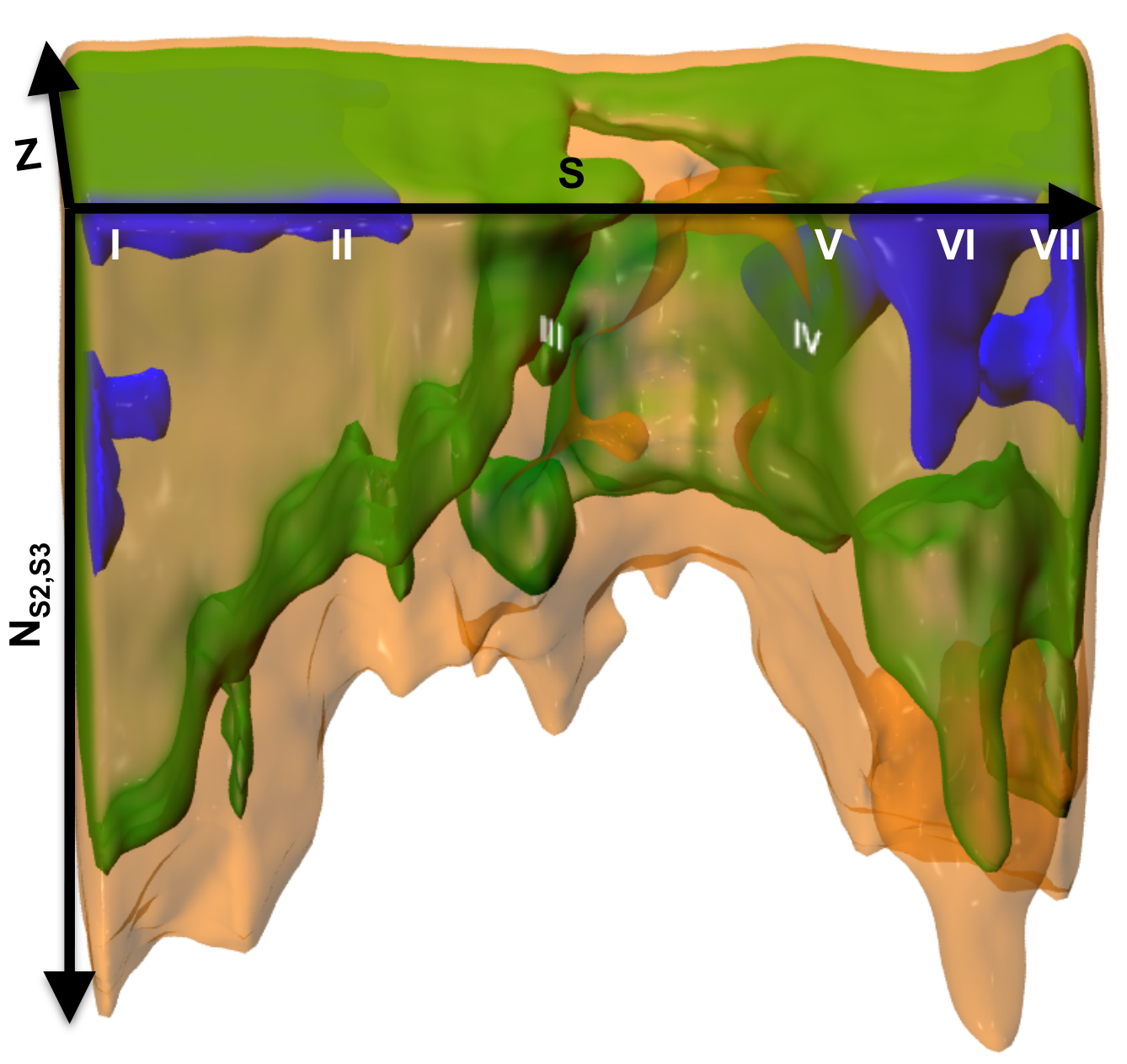


**Figure S2.** **3D free energy.** 3D contour plot describing the free energy of ion permeation along the chosen collective variables. The labels correspond to the basins shown in Figure 4A and to the configurations of Figure 4B.

**Parameterization of the Brownian Dynamics model**

Here we discuss the parameters and the procedures used to perform the Brownian Dynamics (BD) simulations in order to calculate the channel conductance. The BD simulations were carried out within the framework of a kinetic Monte Carlo algorithm, were the position of the ions were assigned to discrete states of a continuous-time Markov chain. All the transitions among the states were modeled as first-order processes, and the corresponding survival times were generated by an exponentially distributed random number. We refer the reader to [1] for details concerning the original derivation of the method.

To perform BD simulations, the transition rates for the forward and backward motions of ions inside the filter, and the entry/exit rates (either from the intra- or the extra-cellular side) of ions into/from the filter, must be defined [1]. To do so, four basic ingredients are required: *i*) the free energy of ion motion expressed as a function of the *z*-position of the three ions, *F*(*z_1_*, *z_2_*, *z_3_*), *ii*) the transmembrane electric potential profile, *iii*) the diffusion constant profile of ions into the filter, and *iv*) the ion concentration on the intra- and extra-cellular side.

1. **Free energy of ion motion *F*(*z_1_*, *z_2_*, *z_3_*)**

Since the free energy of ion motion was calculated essentially as a function of Path Collective Variables (PCVs [2], the third variable *N_S2,S3_* was used to map the conformations of the filter along the ions’ translocation), a histogram reweighting scheme was required to extract the three-dimensional information of ion motion as a function of (*z_1_*, *z_2_*, *z_3_*) from the 2D description (*S*, *Z*).

First, we focused on a slice of the 3D free energy (*F*(*S*, *Z, N_S2,S3_*)) describing the lowest energy pathway for both the mechanisms observed, so to eliminate the unnecessary regions of the FES corresponding to high energy conformational states of the filter. The corresponding states visited along the metadynamics simulation, which were traced during the run with a high saving frequency by exploiting a standard output function of the PLUMED-1.2.2 plugin [3], were pruned too. After that, the resulting 2D FES (*F*(*S*, *Z*)) was binned in the (*S*, *Z*) space, whereas the file containing the record of the visited configurations was binned both in (*S*, *Z*) and in (*z_1_*, *z_2_*, *z_3_*), so to obtain a one-to-one mapping of the two spaces. Assuming a flat free energy surface as it would be obtained in an ideally converged metadynamics simulation, the probability distribution in (*S*, *Z*) was calculated as:

$$w(S,Z)=e^{-\frac{1}{k_{B}T}F(S,Z)}/n(S,Z)$$

where *k_B_* and *T* have the usual meanings, whereas *n*(*S*, *Z*) is a normalization factor which counts all the configurations in the considered bin. Then, the free energy as a function of (*z_1_*, *z_2_*, *z_3_*) was recovered by the reweighted probability obtained by summing *w*(*S*, *Z*) to all the visited states in the corresponding (*z_1_*, *z_2_*, *z_3_*) bin:

$$F(z_{1},z_{2},z_{3})=-k_{B}T\ln(\sum_{z_{1},z_{2},z_{3}} w(S,Z))$$

The resulting *F*(*z_1_*, *z_2_*, *z_3_*) was employed to parameterize the transition rates as reported in [1].

1. **Electric potential profile**

The free energy obtained with the atomistic simulations was calculated in equilibrium conditions, corresponding to a null electrochemical potential. To apply an external driving force on the permeating ions in the BD model, the free energy was shifted in such a way to simulate an electrical potential drop over the membrane according to [1,4]:

$$F_{tot}(z_{1},z_{2},z_{3})=F_{eq}(z_{1},z_{2},z_{3})+\sum_{i=1}^{3} q\Phi_{TM}(z_{i})$$

where *q* is the charge of the ion, and *Ф_TM_* is the electrostatic potential. The input transmembrane potential was parameterized to linearly decay between the SF extremities (*z* = 4 – 16 Å).

1. **Diffusion constant profile**

An accurate estimation of the diffusion coefficient profile would require the calculation of the velocity autocorrelation function of the permeating ions [1]. Here, we rather followed an empirical approach: we used the experimental value of 0.185 Å^2^ ps^-1^ ([1] and references therein) for the ions in the bulk, whereas a relative diffusion constant of 0.8 was used for the ions inside the filter.

1. **Ion concentration**

In line with single channel conductance experiment, the driving force of the BD simulations was primarily controlled by the transmembrane electrostatic potential, whereas symmetric K^+^ concentrations were maintained at the intra- and extra-cellular sides of the model system. The BD simulations were therefore performed at a symmetric potassium concentration of 100 mM.

A cross-sectional area for the filter vestibule of 30 Å^2^ was used. Whenever one ion exited the filter, leaving only 2 ions inside the simulation model, the free energy was calculated as a Boltzmann average between *F*(*z_1_*, *z_2_*, *z_3_*) and *F*(*z_1_*, *z_2_*). The latter free energy difference was empirically tuned so to achieve an average occupancy ratio (2/3 ions) of the filter of about 0.5 [1].

**References**

1. Bernèche S, Roux B (2003) A microscopic view of ion conduction through the K+ channel. Proc Natl Acad Sci USA 100: 8644-8648.

2. Branduardi D, Gervasio FL, Parrinello M (2007) From A to B in free energy space. J Chem Phys 126: 054103.

3. Bonomi M, Branduardi D, Bussi G, Camilloni C, Provasi D, et al. (2009) PLUMED: A portable plugin for free-energy calculations with molecular dynamics. Comput Phys Comm 180: 1961-1972.

4. Roux B (1999) Statistical Mechanical Equilibrium Theory of Selective Ion Channels. Biophys J 77: 139-153.
